# Body Mass Index and Its Influence on Electrocardiographic Parameters in Healthy and Cardiovascular Patients

**DOI:** 10.1002/osp4.70107

**Published:** 2025-12-16

**Authors:** Roholla Hemmati, Mohanna Parvenous, Dorsa Bahrami Zanjanbar, Seyed Hossein Kia, Abbas Soleimani, Sina Bakhshaei

**Affiliations:** ^1^ Department of Cardiology School of Medicine Ilam University of Medical Sciences Ilam Iran; ^2^ School of Medicine Iran University of Medical Sciences Tehran Iran; ^3^ Pharmaceutical Science Research Center, Tehran Medical Sciences Islamic Azad University Tehran Iran; ^4^ GI Pharmacology Interest Group (GPIG) Universal Scientific Education and Research Network (USERN) Tehran Iran; ^5^ Mostafa Khomeini Hospital Cardiovascular Research Center School of Medicine Ilam University of Medical Sciences Ilam Iran; ^6^ Cardiovascular Fellow UHS Southwest Healthcare MEC Temecula California USA

**Keywords:** BMI, electrocardiogram, PR interval, QRS interval, QT interval

## Abstract

**Objective:**

Cardiovascular diseases (CVDs) are a significant cause of mortality worldwide. Smoking, unhealthy diets, physical inactivity, and psychological stress are the main lifestyle factors associated with CVDs. This study was designed to investigate the relationship between electrocardiographic (ECG) changes and various body mass index (BMI) categories, considering demographic characteristics of the participants, such as age, gender, height, and weight.

**Methods:**

This cross‐sectional study was conducted on 964 patients attending Mostafa Khomeini Hospital, Ilam, Iran. Pearson correlation coefficients and regression models were employed using SPSS v. 24 to assess the relationship between BMI and ECG parameters recorded using a 12‐lead ECG.

**Results:**

A 10‐cm increase in height (165 ± 14) was associated with a 0.089 ms decrease in the PR interval, while a 5‐kg weight gain resulted in a 0.20 ms increase in the PR interval (*p* = 0.49). The QRS interval increased by 2 and 0.03 ms, with a 10 cm height and 5 kg weight gain, respectively. The QT interval remained stable with changes in height but decreased by 1.34 ms with a 5‐kg increase in weight.

**Conclusion:**

The findings of this study suggest that ECG parameters are influenced by BMI and may be related to cardiovascular risks. An increase in PR and QRS intervals, a decrease in QRS voltage, and alterations in the ST segment and QT interval underscore the importance of maintaining a healthy BMI to reduce the risk of CVD. This message encourages proactive lifestyle changes to improve heart health.

**Abbreviations**
AHA: American Heart Association
BMI: body mass index
CVDs: cardiovascular diseases
cm: centimeter
ECG: electrocardiogram
ESC: European Society of Cardiology
kg: kilograms
mm: Millimeter
ms: milliseconds
mV: millivolts
SD: Standard deviation
WHO: World Health Organization

## 1 Introduction

The World Health Organization (WHO) recognizes cardiovascular disease (CVD) as the leading cause of death among all disease causes, with mortality rates significantly increasing with age. Although CVD manifests as heart attacks and strokes, symptoms often do not appear until later in life. Typically, risk factors for this disease begin to emerge during childhood and adolescence [1]. In recent years, the prevalence of CVD has increased in many developing countries due to an aging population and lifestyle changes, including high stress levels, mental health issues, smoking, alcohol consumption, physical inactivity, and poor dietary habits, posing a serious threat to public health [2, 3]. According to the WHO reports, CVD accounts for more than three‐quarters of deaths that could be prevented through appropriate lifestyle changes [4]. Obesity is one of the main risk factors contributing to CVD. Numerous studies have found a relationship between electrocardiogram (ECG) changes and mortality rates associated with these factors [5, 6, 7, 8, 9]. Obesity was linked to a variety of ECG changes, such as deviation in the left axis, alterations in the P‐wave, decreased QRS voltage, left ventricular hypertrophy, T‐wave flattening, and prolongation of the QT and QTc intervals.

These changes may indicate an elevated risk of atrial fibrillation and sudden cardiac death [10, 11].

Adipose tissue around the chest wall and heart can physically displace the heart, causing a leftward shift of the electrical axis seen as left axis deviation on ECG. Increased blood volume and pressure in obesity enlarge the left atrium, leading to prolonged P‐wave duration and changes in P‐wave morphology. Excess adipose tissue around the heart attenuates electrical signals and reduces QRS voltage. The increased workload on the left ventricle may cause left ventricular hypertrophy, reflected as increased R‐wave amplitude on ECG. Moreover, obesity‐induced myocardial repolarization abnormalities and electrolyte imbalances contribute to T‐wave flattening and prolongation of QT and QTc intervals, which are linked to higher risks of atrial fibrillation and sudden cardiac death [11, 12].

Beyond structural and electrophysiological effects, obesity exerts a chronic systemic inflammatory state that accelerates vascular injury and myocardial remodeling. Elevated levels of circulating interleukin‐6 (IL‐6), tumor necrosis factor‐alpha (TNF‐α), and C‐reactive protein (CRP) have been documented in obese individuals, and these biomarkers are associated with endothelial dysfunction and impaired cardiac conduction [13]. In addition, adipokines such as leptin and adiponectin, secreted by adipose tissue, play dual roles in modulating cardiac metabolism and arrhythmia susceptibility. Leptin excess has been linked to sympathetic activation, while reduced adiponectin contributes to impaired cardioprotection and metabolic stress [13, 14].

Importantly, the relationship between obesity and cardiovascular disease is not limited to structural cardiac changes. Recent studies suggest that obesity alters autonomic nervous system balance, shifting toward increased sympathetic activity and reduced vagal tone. This autonomic imbalance may contribute to arrhythmogenesis and has been associated with prolonged PR and QRS intervals in obese populations. Furthermore, obesity‐related sleep disorders, such as obstructive sleep apnea, exacerbate hypoxemia and oxidative stress, further impairing myocardial electrophysiology. These multifactorial interactions highlight the complexity of obesity as both a direct and indirect driver of cardiovascular morbidity [15, 16, 17].

Genetic and epigenetic factors further complicate the relationship between obesity and cardiac abnormalities. Polymorphisms in genes regulating ion channels, myocardial structure, or adipose tissue metabolism may predispose obese individuals to specific ECG patterns or arrhythmias. Epigenetic modifications, such as DNA methylation linked to high‐fat diets, have also been shown to influence cardiac electrophysiology. These insights point toward a precision medicine approach, where BMI alone may be insufficient, and genetic background and metabolic phenotype must also be considered in risk stratification [18, 19, 20].

By addressing the gap in existing research, this study attempted to investigate the relationship between ECG abnormalities and various BMI classifications, as well as their associations with different demographic characteristics (age, gender, height, and weight) of the participants. The present study seeks to enhance the understanding of how obesity‐related ECG changes correlate with specific BMI categories and demographic factors.

## 2 Materials and Methods

This cross‐sectional study was conducted among patients attending Mostafa Khomeini Hospital in Ilam City (Ilam, Iran), a region predominantly inhabited by the Kurdish population in Iran. Over a 3‐month period, a list of all individuals aged ≥ 18 years attending the hospital was generated. From this list, 964 eligible participants were randomly selected using a random‐number table (simple random sampling) and were classified into three groups: healthy individuals, patients with a history of cardiovascular diseases (CVDs), and patients taking cardiovascular medications.

The demographic characteristics of the participants, especially age and gender, were recorded in a checklist based on their medical records. Body weight (kg) and height (cm) were measured using standardized equipment. Based on BMI, the participants were categorized into four classes: Group 1 (BMI < 18.5), Group 2 (18.5 ≤ BMI < 24.9), Group 3 (25 ≤ BMI < 29.9), and Group 4 (BMI ≥ 30).

ECG parameters were recorded following anthropometric measurements and a 10‐min resting period. During this time, participants were positioned supine and were asked to refrain from eating for 1 h before the test. Standard 12‐lead ECGs were then recorded using the Philips Page Writer TC70 electrocardiograph (Philips Healthcare, Andover, MA, USA). Recordings were made at a paper speed of 25 mm/s with a calibration of 10 mm/mV. A high‐pass filter at 0.05 Hz, a low‐pass filter at 150 Hz, and a 50 Hz notch filter were applied to reduce baseline wander and electrical interference. For each participant, at least three consecutive cardiac cycles per lead were recorded, and measurements were taken from the median complex. Special attention was given to minimize ECG artifacts, particularly in participants with obesity, where excess adipose tissue and chest wall thickness could interfere with electrode contact and signal quality. To address this, electrode sites were cleaned and, if necessary, lightly abraded to reduce skin impedance, and adhesive gel electrodes were used to ensure stable contact. Consistent electrode placement was maintained according to the AHA/ESC guidelines, and recordings with obvious motion artifacts or poor signal quality were repeated.

All ECGs were independently interpreted by two blinded cardiologists, and in cases of disagreement, consensus was reached through joint review and discussion between the same two cardiologists.

The inter‐observer agreement for ECG parameters (PR, QRS, QT intervals, QRS voltage, and ST‐segment changes) was assessed using Cohen's kappa statistic, which demonstrated substantial agreement (*κ* = 0.82).

Participants were instructed to breathe normally and refrain from speaking during the procedure. ECG parameters, including QRS voltage and duration, PR interval, QT interval, and ST‐segment changes, were captured at a speed of 25 mm per second and with a sensitivity of 10 mV per mm, and were validated by cardiac specialists. Participants were subsequently categorized into two groups based on their medical history and were included in the final analysis of the study. The case group comprised patients with a documented history of cardiovascular diseases (CVDs). This group was determined based on echocardiography, clinical symptoms, radiography, medication use, coronary artery disease, valve disease, cardiomegaly, or pericarditis. The healthy group comprised patients with no history of cardiovascular disease, no cardiovascular medication use, and no evidence of cardiovascular pathology in their medical records. No additional imaging (e.g., echocardiography, angiography) was performed to exclude subclinical disease.

Changes in QRS voltage were assessed using ECG guidelines provided by the American Heart Association (AHA) and the European Society of Cardiology (ESC). Normal QRS voltages were observed as ≤ 2.5 mV in the limb leads and ≤ 3.0 mV in the precordial leads. In contrast, low QRS voltage was defined as > 0.5 mV in the limb leads and 1.0 mV in the precordial leads, as compared to the baseline ECGs. ST‐segment deviations were assessed using the criteria established by the AHA and ESC. Significant deviations were defined as ST elevation of at least 1 mm (0.1 mV) at the J‐point in two or more contiguous limb leads or at least 2 mm (0.2 mV) in precordial leads for men aged ≥ 40, 2.5 mm (0.25 mV) for men < 40, and 1.5 mm (0.15 mV) for women. ST‐segment depression was defined as a horizontal or down‐sloping ST depression of at least 0.5 m (0.05 mV) at the J‐point in two or more contiguous leads. Additionally, an upsloping ST‐segment depression of 1 mm (0.1 mV) was considered significant when it was accompanied by relevant clinical symptoms and other ECG abnormalities. To minimize external factors that could affect the accuracy of the results, we ensured consistent electrode placement, recorded ECGs with patients in the supine position, performed multiple measurements for accuracy, correlated any ECG changes with the patients' clinical findings, and excluded individuals with conditions known to interfere with ECG accuracy. These measures were performed to ensure that the assessments of QRS voltage changes are accurate and clinically meaningful.

The inclusion criteria of the study entailed participants aged 18 years or older who gave their verbal consent and had complete and accurate medical records. Healthy individuals, cases with a history of CVDs, and those taking cardiovascular medications were also included in the study. Exclusion criteria included participants with incomplete medical records, acute medical conditions that may affect ECG accuracy, lack of consent, pregnancy, significant noncardiac conditions that influence measurements, technical issues with ECG devices, or physical limitations that prevent accurate height and weight measurements. Participants who refused to participate in the study were also excluded. Data were entered into SPSS version 24 for statistical analysis. In addition to obtaining verbal consent from the patients, ethical approval was obtained from the Ethics Committee of Ilam University of Medical Sciences.

## 3 Result

### 3.1 Demographic Information

Demographic characteristics of the participants are summarized in Table 1. According to the WHO classification, participants were categorized into four BMI groups. The distribution of BMI categories and demographic data, including age, gender, weight, and height, is detailed in the table. The healthy group included 484 individuals (230 males and 254 females), while the case group included 480 individuals (195 males and 285 females). Further details on weight, height, age, and BMI distributions are presented in Table 1.

**TABLE 1:** Demographic information of the participants.

Characteristics	Total participants (*N* = 964)	Healthy group (*N* = 484)	Case group (*N* = 480)
Number of participants	964	484	480
Male	425 (44.1%)	230 (47.5%)	195 (40.6%)
Female	539 (55.9%)	254 (52.5%)	285 (59.4%)
Age range (years)	18–91	18–91	21–88
Mean age (years)	54.74 ± 15.68	47 ± 8	62 ± 8
Weight range (kg)	38–133	38–133	38–121
Mean weight (kg)	74 ± 27	76 ± 27	71 ± 24.9
Height range (cm)	143–200	143–200	145–191
Mean height (cm)	166 ± 10	168 ± 16	164 ± 14
BMI range (kg/m^2^)	15–46	16–43	15–46
Mean BMI	22.17 ± 3.82	26.1 ± 7.8	26 ± 8
BMI categories in healthy group:			
‐ Category 1 (underweight)	17 (1.8%)	10 (2%)	7 (1.4%)
‐ Category 2 (normal weight)	343 (35.6%)	154 (31.8%)	189 (39.4%)
‐ Category 3 (overweight)	403 (41.8%)	216 (44.6%)	187 (38.9%)
‐ Category 4 (obese)	201 (20.8%)	104 (21.6%)	97 (20.3%)

*Note:* This table summarizes the demographic data of the participants in the healthy and case groups, including gender distribution, age, weight, height, and body mass index (BMI). Mean values with standard deviations are provided for each parameter.

### 3.2 PR Interval

The PR interval averaged 164 ± 27 ms in males and 162 ± 25 ms in females, with a statistically significant difference observed between genders (Table 2). This measure decreased by 0.089 ms, with a 10‐cm increase in height (mean 165 ± 14 cm). Notably, in the healthy group, the PR interval decreased by 0.03 ms with a 10‐cm increase in height; in the case group, it increased by 2 ms. Both of these changes were statistically significant (*p* < 0.05). Furthermore, in the case group, the PR interval increased by 0.20 ms with a 5‐kg weight gain, though this change was not statistically significant (*p* = 0.49). In both the healthy and case groups, the PR interval increased by 0.76 ms (*p* = 0.04) and 0.20 ms (*p* = 0.65), respectively (Table 3). The highest mean PR interval recorded was 175 ± 35 ms in the case group in the first BMI group (BMI < 18.5). In contrast, in the healthy group, it was 163 ± 22 ms, associated with the fourth group (BMI > 30), indicating a statistically significant difference (Table 4).

**TABLE 2:** Relationship of gender with PR, QRS, and QT interval.

Parameters (ms)	Gender	Number	Average (ms)
PR interval	Male	425	164 ± 27
	Female	539	162 ± 25
QRS interval	Male	425	107 ± 17
	Female	539	98 ± 3
QT interval	Male	425	381 ± 33
	Female	539	378 ± 33

*Note:* This table presents the average values of PR, QRS, and QT intervals among male and female participants, along with the number of individuals in each gender group.

**TABLE 3:** Mean PR/QRS/QT interval and its correlation with every 10 cm increase in height and 5 kg weight increase in the two study groups.

Population	Mean		Interval changes	*p* value
PR interval (ms)				
Healthy group	Increase per 10 cm height	158 ± 18	0.03 ms increase	0.000
	Increase per 5 kg weight	147 ± 6	0.76 ms increase	0.04
Case group	Increase per 10 cm height	131 ± 22	2 ms increase	0.000
	Increase per 5 kg weight	165 ± 6	0.20 ms increase	0.65
Total	Increase per 10 cm height	165 ± 14	0.08 ms decrease	0.000
	Increase per 5 kg weight	160 ± 4	0.20 ms increase	0.49
QRS interval (ms)				
Healthy group	Increase per 10 cm height	45 ± 9	3 ms increase	0.000
	Increase per 5 kg weight	95 ± 3	0.36 ms increase	0.08
Case group	Increase per 10 cm height	40 ± 14	3 ms increase	0.000
	Increase per 5 kg weight	105 ± 4	0.11 ms decrease	0.70
Total	Increase per 10 cm height	52 ± 8	2 ms increase	0.000
	Increase per 5 kg weight	101 ± 2	0.03 ms increase	0.84
QT interval (ms)				
Healthy group	Increase per 10 cm height	394 ± 22	1 ms decrease	0.000
	Increase per 5 kg weight	396 ± 7	1.41 ms decrease	0.00
Case group	Increase per 10 cm height	329 ± 28	3 ms increase	0.04
	Increase per 5 kg weight	394 ± 8	0.66 ms decrease	0.24
Total	Increase per 10 cm height	386 ± 17	No change	0.000
	Increase per 5 kg weight	399 ± 5	1.34 ms decrease	0.00

*Note:* This table reports the mean values of PR, QRS, and QT intervals in relation to every 10 cm increase in height and every 5 kg increase in weight, analyzed separately in healthy, case, and total populations. *p*‐values indicate the significance of the observed changes.

**TABLE 4:** PR interval mean in different BMI groups and their frequencies.

BMI group	PR interval	
	Healthy group	Case group
1	Frequency: 10	Frequency: 7
	Mean: 148 ± 32	Mean: 175 ± 35
2	Frequency: 154	Frequency: 189
	Mean: 155 ± 23	Mean: 169 ± 31
3	Frequency: 216	Frequency: 187
	Mean: 159 ± 25	Mean: 167 ± 25
4	Frequency: 104	Frequency: 97
	Mean: 163 ± 22	Mean: 166 ± 27
*p* value	0.05	0.00
Total	Frequency: 484	Frequency: 480

*Note:* This table shows the frequency and average PR interval (± SD) across four BMI groups in both healthy and case participants. *p*‐values denote statistical significance.

### 3.3 QRS Interval

The average QRS interval was 107 ± 17 ms in males and 98 ± 13 ms in females (Table 2). This measure increased by 2 ms with every 10 cm increase in height (52 ± 8 cm). In the healthy group, the QRS interval increased by 3 ms, with a 10 cm increase in height. It also increased by 3 ms in the case group (*p* = 0.000; Table 3). Moreover, the QRS interval increased by 0.03 ms with every 5 kg increase in weight (*p* = 0.84). In the healthy group, it increased by 0.36 ms (*p* = 0.08), while in the case group, it decreased by 0.11 ms (*p* = 0.70; Table 3).

### 3.4 QT Interval

The mean QT intervals in males and females were 381 ± 33 and 378 ± 33, respectively (Table 2). The mean QT interval remained unchanged with a 10 cm increase in height (386 ± 17 ms). In the healthy group, this measure decreased by 1 ms with a 10 cm increase in height, whereas in the case group, it increased by 3 ms (*p* = 0.04). Additionally, the QT interval decreased by 1.34 ms with a 5 kg increase in overall weight. In both the healthy and case groups, it was also reduced by 1.41 and 0.66 ms in the healthy and case groups, respectively (*p* = 0.024; Table 3).

### 3.5 QT Interval in Different BMI Groups

Table 5 presents the mean QT intervals across different BMI groups in the total population, as well as in the healthy and case subgroups. No significant correlation was found between the QT interval and BMI groups in the overall population (*p* = 0.06). However, a statistically significant association was observed in both the healthy and case groups (*p* = 0.000 for each).

**TABLE 5:** Frequency and mean of the QT interval in different BMI groups.

BMI QT interval	Healthy group	Case group	Total
Group 1 frequency	10	7	17
Mean	381 ± 35	390 ± 29	385 ± 32
Group 2 frequency	154	189	343
Mean	377 ± 32	387 ± 37	383 ± 35
Group 3 frequency	216	187	403
Mean	372 ± 26	386 ± 32	379 ± 30
Group 4 frequency	104	97	201
Mean	373 ± 35	377 ± 35	375 ± 35
*P*	0.000	0.000	0.06
Total	484	480	964

*Note:* This table presents the frequency and average QT interval in different BMI groups for healthy, case, and total populations, along with associated *p*‐values.

### 3.6 QRS Voltage in Different BMI Groups

Among the 799 individuals studied, the distribution of QRS voltage states across genders is summarized in Table 6. Most details, including the number and percentage of each QRS voltage categories by gender, are provided in Table 6. Also, all 799 individuals were assessed for QRS voltage and anthropometric measurements. In the overall population, individuals with normal QRS voltage had an average height of 166 ± 10 cm and a weight of 74 ± 14 kg. Those with decreased and increased QRS voltages had an average height of 165 ± 8 cm and 166 ± 9 cm, and an average weight of 71 ± 16 kg and 70 ± 12 kg, respectively. A statistically significant relationship was found between QRS voltage and participants' height and weight.

**TABLE 6:** Relationship between gender and QRS voltage and ST segment.

	Gender	Overall number	State
QRS voltage	Male	425 (44.1%)	Low: 6 (40%)
			Normal: 340 (43%)
			High: 79 (53%)
	Female	539 (55.9%)	Low: 9 (60%)
			Normal: 459 (57%)
			High: 71 (47%)
ST segment	Male	425 (44.1%)	Normal: 337 (43%)
			Non Normal: 88 (46%)
	Female	539 (55.9%)	Normal: 438 (57%)
			Non normal: 101 (54%)

*Note:* This table illustrates the distribution of QRS voltage levels (low, normal, high) and ST segment status (normal vs. non‐normal) by gender.

In the healthy group, all 410 individuals with normal QRS voltage had an average height of 168 ± 10 cm and a weight of 76 ± 14 kg. Those with decreased and increased QRS voltage (*n* = 4 and *n* = 70, respectively) had an average height of 166 ± 8 cm and 170 ± 10 cm, and weights of 82 ± 18 kg and 73 ± 13 kg. This association between QRS voltage and anthropometric parameters was statistically significant.

In the case group, individuals with normal QRS voltage (*n* = 389) had an average height of 164 ± 9 cm and a weight of 72 ± 14 kg. Those with decreased and increased QRS voltage (*n* = 11 and *n* = 80) had an average height of 164 ± 9 cm and 163 ± 8 cm, and weight of 67 ± 13 kg and 67 ± 11 kg, respectively. A significant association was also observed in this group.

The distribution of QRS voltage across BMI groups in both healthy and case populations is provided in Table 7.

**TABLE 7:** Prevalence of BMI in different QRS voltages in healthy, case, and total groups.

QRS voltage										
BMI group		Total			Healthy group			Case group		
		Normal	Low	High	Normal	Low	High	Normal	Low	High
1	Frequency	13	0	4	7	0	3	6	0	1
	Percentage	1	0	2	1	0	4	1	0	1
2	Frequency	267	8	68	124	1	29	143	7	39
	Percentage	33	53	45	31	25	41	37	64	49
3	Frequency	342	4	57	186	2	28	156	2	29
	Percentage	43	26	39	45	50	41	41	18	37
4	Frequency	177	3	21	93	1	10	84	2	11
	Percentage	23	21	14	23	25	14	21	18	13
	Total	799	15	150	410	4	70	389	11	80
	Percentage	100	100	100	100	100	100	100	100	100

*Note:* This table displays the frequency and percentage of participants in each BMI group according to QRS voltage classification (low, normal, high), stratified by healthy, case, and total populations.

### 3.7 ST Segment in Different BMI Groups

Among the 799 individuals, 775 had normal ST segments, and 189 showed abnormalities. The distribution of ST‐segment status by gender is presented in Table 6. Among the total population, 775 individuals exhibited regular ST segments, with an average height of 166 ± 10 cm and a weight of 75 ± 14 kg. Additionally, 181 individuals had depressed ST segments (164 ± 9 cm height, 69 ± 13 kg weight), and 8 individuals had elevated ST segments (173 ± 5 cm height, 63 ± 9 kg weight).

In the healthy group, individuals with normal, depressed, and elevated ST segments had average heights of 169 ± 10 cm, 167 ± 10 cm, and 175 ± 9 cm, and average weights of 77 ± 14 kg, 72 ± 14 kg, and 68 ± 14 kg, respectively.

In the case group, individuals with normal, depressed, and elevated ST segments had average heights of 164 ± 9 cm, 163 ± 8 cm, and 172 ± 2 cm, and average weights of 73 ± 14 kg, 67 ± 12 kg, and 60 ± 6 kg, respectively.

Further details regarding the distribution of ST‐segment patterns across BMI groups in the total population, healthy individuals, and diseased individuals are summarized in Table 8.

**TABLE 8:** Frequency of BMI in ST‐segment changes in healthy, case, and total groups.

ST segment										
BMI group		Total population			Healthy individuals			Diseased individuals		
		Normal	Low	High	Normal	Low	High	Normal	Low	High
1	Frequency	10	6	1	6	4	0	4	2	1
	Percentage	1	3	13	1	6	0	1	1	20
2	Frequency	258	78	7	131	20	3	127	58	4
	Percentage	33	44	87	31	33	100	36	49	80
3	Frequency	326	77	0	186	30	0	140	47	0
	Percentage	43	42	0	45	49	0	39	40	0
4	Frequency	181	20	0	97	7	0	84	13	0
	Percentage	23	11	0	24	12	0	24	10	0
	Total	775	181	8	420	61	3	355	120	5
	Percentage	100	100	100	100	100	100	100	100	100

*Note:* This table presents the frequency and percentage of individuals in each BMI group in relation to normal and abnormal ST segments, categorized by healthy, case, and total populations.

## 4 Discussion

The present study investigated the association of ECG alterations with different BMIs in two groups. The mean PR, QRS, and QT intervals were significantly lower in females than in males. Among 964 participants randomly included in the final analysis, 425 individuals were males (44.1%), and 539 were females (55.9%). The participants were divided into healthy (230 males and 254 females) and case (195 males and 285 females) groups In the studies by Zuppiolo et al. and Mohseni et al., more females than males were included [21, 22], and since gender influences ECG parameters, comparing this study's results with those of the previous studies is likely to yield similar findings regarding gender prevalence.

The results of the present study demonstrated a significant relationship between the PR intervals (164 ± 27 in males and 162 ± 25 in females) and genders. However, in the study conducted by Suthar et al., no correlation was found between age, gender, BMI, and ECG parameters, likely due to the small sample size. They also indicated that healthy obese individuals exhibited a higher prevalence of abnormal ECG findings compared with healthy individuals with normal weight [23]. Alidousti et al. found a significantly higher mean BMI in the case group than in the control group, and obese individuals had more AMI events. Central obesity was more strongly associated with acute myocardial infarction than general obesity [24]. Similarly, a recent study from the Kailuan Cohort demonstrated that obesity and physical inactivity are associated with an increased risk of cardiac conduction disorders, including atrioventricular block and potential alterations in PR interval, supporting our findings of increased PR interval in individuals with higher BMI [25]. Therefore, studies indicate that obesity increases the risk of heart disease.

In this study, the overall PR interval increased by 0.20 ms, with a 5 kg increase in weight (*p* = 0.49). This elevation was primarily observed in the healthy group, which experienced an increase of 0.76 ms (*p* = 0.04), compared to the case group, which showed an increase of 0.20 ms (*p* = 0.65). However, both Frank et al. and Pipberger et al. found a negligible relationship between PR interval and weight gain, indicating a minimal impact of weight gain on PR interval duration [10, 26]. Additionally, Fraley et al. demonstrated that obesity can also contribute to an increase in the PR interval [11]. The average PR interval was significantly higher in the case group than in the healthy group. Across the total population and within both groups, an increase in BMI was associated with a statistically significant rise in mean PR interval, suggesting a positive correlation between BMI and PR interval length. In contrast, Suthar et al.’s study found that although the PR interval increased in the BMI over 30 groups, the change was not statistically significant [23].

Based on our results, the QRS interval increased by 0.03 ms with a weight gain of 5 kg (*p* = 0.84). In the healthy group, this measure increased by 0.36 ms (*p* = 0.08), while in the case group, it decreased by 0.11 ms (*p* = 0.70). In the study by Suthar et al., although the QRS interval increased in individuals with a BMI over 30, this change was not statistically significant [23]. Similarly, Frank et al. reported a minimal relationship between the QRS interval and the percentage increase in weight, which was not clinically significant [10]. In contrast, significant differences were observed in the QRS interval between the healthy and case groups, with an average of 105 ± 13 ms in the healthy group and 113 ± 22 ms in Group 1 of the case group. This indicates that individuals in the case group had longer QRS intervals on average. A statistically significant association between BMI and QRS interval was also found in both groups, suggesting that BMI contributes to QRS interval differences. Additionally, Murkofsky et al. reported a statistically significant prolongation of the QRS interval in patients with an ejection fraction < 45% compared to those with > 45% [27]. Similarly, a recent large cohort study on more than 450,000 participants demonstrated a significant association between obesity and conduction abnormalities, including prolongation of the QRS interval. These findings further support the role of increased BMI in cardiac conduction changes [28].

The current study found a statistically significant decrease of 1.34 ms in the QT interval with each 5 kg increase in weight across the total population (*p* = 0.000). In the healthy and case groups, the QT interval decreased by 1.41 ms (*p* = 0.000) and 0.66 ms (*p* = 0.024), respectively. A significant relationship was also observed between the QT interval and BMI groups. Moreover, the average QT interval was significantly higher in the case group than in the healthy group. In contrast, Suthar et al. reported increases in the QT and QTc intervals in individuals with a BMI over 30, but these changes were not statistically significant [23]. In a study conducted by Girola and colleagues, the QTc interval was lower in overweight individuals than in those with normal weight, though this difference was not statistically significant [29], which aligns with the present study. Furthermore, the survey by Nomura et al. suggested a decrease in QTc in overweight groups compared with those with normal weight [1]. However, some studies have reported an increased QTc interval in overweight groups [10, 11, 30, 31, 32, 33], which contrasts with our results. Consistently, a recent study demonstrated that individuals with obesity exhibited prolonged QTc intervals and increased Tpeak‐Tend interval duration, with these alterations being positively associated with BMI and waist circumference, further supporting the notion that obesity may contribute to abnormal ventricular repolarization [34]. The discrepancies observed across studies may be explained by several factors. First, Study‐related characteristics such as sample size, population differences, and measurement techniques can influence the findings, while measurement errors may further contribute to inconsistencies. Second, individual and biological factors play a role; for instance, body fat distribution and sex‐specific associations (e.g., central adiposity) may produce distinct electrophysiological outcomes. In addition, genetic or environmental influences tied to ethnicity or population background can modulate ventricular repolarization responses to adiposity. Third, methodological issues such as measurement artifacts in obese individuals, differential medication use, electrolyte disturbances, and sample composition (including the proportion of CVD cases) may bias the observed associations. Finally, given the conflicting evidence in the literature—ranging from reports of QT prolongation in obesity to QT shortening after weight loss, along with critiques of earlier findings—we interpret our results with caution and recommend further adjusted and longitudinal analyses to clarify causality.

There was a correlation between QRS voltage and anthropometric measures such as height and weight. Individuals with greater body weight showed lower QRS voltages, suggesting a physiological link between increased adiposity and reduced cardiac electrical activity. These findings highlight the need to consider body composition when interpreting QRS voltage. Similarly, Fraley et al. found that obesity, especially with comorbidities, was associated with decreased QRS voltage [11]. Eisenstein and associates enrolled 144 individuals and, following a 300‐calorie daily diet over 6 months, changes in QRS voltage were observed: 15 patients showed an increase, 37 a decrease, and 6 exhibited no change post‐weight loss [35]. These results are consistent with those of the present study.

Our study found that changes in the ST segment were more prevalent in the case group than in the healthy group, particularly among individuals with higher body weight and shorter height. Additionally, Alpert et al. reported a non‐significant decrease in the ST segment among obese and case groups [36]. Conversely, Suthar observed that 90% of individuals with a BMI over 30 had normal ST segments, compared to 100% in the group with a BMI less than 25, with only 10% of the individuals in the BMI over 30 group exhibiting non‐significant ST segment changes [23].

While previous studies have generally taken a broad approach, this study applies various statistical methods to explore the relationship between BMI, demographic characteristics, and ECG parameters in a large sample of 964 adults. It provides a comprehensive analysis of how BMI categories (four groups) affect ECG readings, including the PR, QRS, and QT intervals. These findings offer valuable insights for clinical practice by aiding in the interpretation of ECG results in overweight and obese patients and potentially helping to prevent cardiovascular complications. However, the study's reliance on a single ECG assessment, and multiple ECG assessments over time could yield deeper insights and reduce false positives. Moreover, our study included both healthy individuals and patients with CVD; the observed associations may reflect both physiological and pathological factors. This introduces potential confounding, which should be considered when interpreting the results. Also, multivariable regression analyses could be performed for future studies to isolate the independent effect of BMI on ECG parameters.

In this study, we did not measure several important lifestyle factors, such as smoking, diet, and physical activity, which could serve as potential confounders in the relationship between BMI and ECG parameters. These factors are known to influence cardiovascular health and could affect the results observed in this study.

For instance, smoking is a well‐established risk factor for cardiovascular disease and can influence ECG findings, while poor dietary habits, particularly high salt or fat intake, could alter heart function and potentially mask or exaggerate the effect of BMI on ECG parameters.

Future studies should aim to include comprehensive data on lifestyle factors to better isolate the effect of BMI on ECG parameters.

Ultimately, identifying obesity‐related ECG changes has clinical importance because it provides an inexpensive and widely accessible screening tool. If validated, BMI‐adjusted ECG parameters could be used in primary care to detect early cardiovascular alterations in asymptomatic obese individuals. This would facilitate timely lifestyle interventions and risk management strategies before overt cardiovascular disease develops.

## 5 Conclusion

Obesity, CVDs, and overweight status are strongly associated with alterations in ECG parameters, including prolonged PR and QRS intervals, changes in QRS voltage, and ST and QT segment variations. Our findings in a relatively large adult population highlight the importance of maintaining a healthy BMI to mitigate these changes and reduce cardiovascular risk. In clinical practice, integrating BMI‐adjusted ECG norms may facilitate the early identification of at‐risk individuals and improve the accuracy of cardiovascular risk stratification. Routine ECG monitoring interpreted in conjunction with BMI status could serve as a cost‐effective and widely accessible screening tool in both primary care and cardiology settings. Moreover, future longitudinal studies are warranted to evaluate whether BMI‐related ECG alterations predict long‐term outcomes such as arrhythmias, myocardial infarction, or sudden cardiac death, thereby reinforcing their utility in preventive cardiology and clinical decision‐making.

## Author Contributions

Roholla Hemmati and Mohanna Parvenous contributed to study design, data collection, and data analysis. Dorsa Bahrami Zanjanbar and Seyed Hossein Kia were involved in data interpretation, literature search, manuscript writing, and conducting the experiments. Abbas Soleimani and Sina Bakhshaei revised the manuscript.

## Funding

The authors have nothing to report

## Conflicts of Interest

The authors declare no conflicts of interest.
